# Report of a Case of Mania

**Published:** 1861-01

**Authors:** F. M. Hunt

**Affiliations:** Liberty Hill, Georgia


### ARTICLE III.

*Report of a case of Mania.* By F. M. Hunt, M. D., of Liberty Hill, Georgia.

A short time since, I was called to see a negro man, aged 27 years. Found him setting in a neat little hut, singing happily and as correctly as might be expected of a negro of ordinary intelligence. When addressed he arose, bowed politely, and expressed himself exceedingly happy. As his mental derangement partook more of the character of religious phrenzy or monomania, from the report of his owner, I directed his attention to that subject, which seemed entirely congenial with his feelings, and about which he talked freely, and soon became much excited; expressing the firm belief in the fact that he would soon be transported from earth without seeing death. When his thoughts were directed to other subjects, he seemed confused, and unable even to answer simple questions correctly.

From his master, the following history was obtained :

Fifteen years ago, he attended the funeral of a negro, and during the exercises he became powerfully excited, and after returning home, seemed absorbed in deep thought, not at all disposed to converse with any one. In a day or two he left his work, sought retirement, and acted strangely in other respects. No effort was made to control him. During the whole day, for several days, he would set alone in the same place, returning home regularly at night. During this time of seclusion his tongue became swollen so as to protrude between his lips, rendering him unable to articulate a word. So soon as this difficulty about the tongue passed of, which lasted only a few days, he went into the maniacal enjoyment of religious exercises. This state soon passed away, and he was found sufficiently rational to attend his ordinary business on the farm. For six or eight years he remained rational and kindly disposed; after this time for about the same number of years, though rational, seemed less inclined to morality. Three months since, he was attacked with erratic pains, affecting the joints particularly. The lumber region seemed to be the seat of considerable pain. In a few days he was relieved, without medication, so as to resume his usual labor on the plantation, but seemed morose and dejected. This state of feeling increased till nothing but religious terror seemed to occupy his mind. This state of mind, three days ago, gave place to great volubility —expressions of pleasure and happiness, praise and exultation. This is the state of mind in which we found him when first called.

Though nothing very unusual is found in this case, it is one which answers well to lead us into the consideration of the true pathology of mania.

The question most important, connected with mental disorders, is, does the mind become deranged by external impressions through the senses, and if so, do these impressions affect the brain itself, originally or functionally? We have in this case, presented evidences of physical disease, doubtless the result of that state of the cerebral nervous centre which directly produced the mania. This is one of the many evidences that might be named in support of the idea that there is organic disease of the brain itself in ordinary mania. And though, as in this case, mental emotions often seem to develop, and always, perhaps, aggravate symptoms of this disease, yet it is questionable whether such influences ever lay the foundation for such change in the brain. The question of mania depending upon organic disease of the brain is a practical one. Many cases of mental derangement in the treatment of which, mental appliances alone are resorted to, might be much more successfully managed by the use of pharmacological agents, directly influencing the physical organism.

Another question, growing out of the history of the case reported above, presents itself, viz: is rheumatic disease dependent upon primary disturbance of the nervous centres? In this case there is an extraordinary coincidence, or a connection between the causes of the mental derangement, and the rheumatic symptoms reported. We are the more ready to concede this connection, since, from the fact that remedies acting exclusively upon the nervous system are more successful in the treatment of rheumatism, we conclude that it originates in nervous disturbance. As the same state of the nervous centres, in this case, in all probability, led to the development of the two forms of disease mentioned, why may not those means found useful in relieving rheumatism in any part of the body relieve the symptoms both of mainia and rheumatism. There is evidently a derangement of the nervous centre, and we have to determine the nature of that physical derangement by the physical manifestation. A perversion of the mental faculties may result from various forms of disease of the brain; plethora, anæmia or any kind of disturbance, modifies or deranges its function, the mind, but the absence or presence of febrile, and other symptoms enable us to settle upon the true condition of the brain. So it is, when rheumatic symptoms are developed in connection with mental alienation; we have a right, at least, to suspicion that form of disturbance of the nervous centres which usually leads to migratory pains called rheumatism. *

* [To carry out an idea which we think a good one, will Dr. Hunt test the effect of quinine in the case reported—*Editor.*]

